# Bioactivity Profiling and Quantification of Gastrodin in *Gastrodia elata* Cultivated in the Field versus Facility via Hyphenated High-Performance Thin-Layer Chromatography

**DOI:** 10.3390/ijms24129936

**Published:** 2023-06-09

**Authors:** Fernanda L. B. Mügge, Cheul Muu Sim, Bernd Honermeier, Gertrud E. Morlock

**Affiliations:** 1 Department of Food Science, Justus Liebig University Giessen, 35392 Giessen, Germany; fernanda.lins-brandao-muegge@ernaehrung.uni-giessen.de; 2 Neutron Science Center, Korea Atomic Energy Research Institute, Daejeon 34057, Republic of Korea; cmsim@kaeri.re.kr; 3 Department of Agronomy and Crop Physiology, Justus Liebig University Giessen, 35392 Giessen, Germany; bernd.honermeier@agrar.uni-giessen.de

**Keywords:** *Gastrodia elata*, planar chromatography, bioanalytical methods, antioxidant, cytotoxicity

## Abstract

*Gastrodia elata* (Orchidaceae) is native to mountainous areas of Asia and is a plant species used in traditional medicine for more than two thousand years. The species was reported to have many biological activities, such as neuroprotective, antioxidant, and anti-inflammatory activity. After many years of extensive exploitation from the wild, the plant was added to lists of endangered species. Since its desired cultivation is considered difficult, innovative cultivation methods that can reduce the costs of using new soil in each cycle and at the same time avoid contamination with pathogens and chemicals are urgently needed on large scale. In this work, five *G. elata* samples cultivated in a facility utilizing electron beam-treated soil were compared to two samples grown in the field concerning their chemical composition and bioactivity. Using hyphenated high-performance thin-layer chromatography (HPTLC) and multi-imaging (UV/Vis/FLD, also after derivatization), the chemical marker compound gastrodin was quantified in the seven *G. elata* rhizome/tuber samples, which showed differences in their contents between facility and field samples and between samples collected during different seasons. Parishin E was also found to be present. Combining HPTLC with on-surface (bio)assays, the antioxidant activity and inhibition of acetylcholinesterase as well as the absence of cytotoxicity against human cells were demonstrated and compared between samples.

## 1. Introduction

*Gastrodia elata* Blume belongs to the family Orchidaceae and is commonly used in traditional medicine. The plant is native to the mountain regions of Eastern Asia, ranging from Nepal to China, Korea, Japan, and Russia [1]. *Gastrodiae rhizoma*, the dried tuber of *G. elata,* is mainly used to treat affections of the central nervous system, such as headache, migraine, dizziness, and epilepsy [2]. Previous scientific works have provided in vitro and in vivo pharmacological evidence of neuroprotective activity [3,4,5,6,7,8,9,10,11], antidepressant [12,13], antioxidant [14,15,16,17,18,19] and anti-inflammatory effects [20,21,22,23].

Unfortunately, overexploitation of the plant in the native regions has placed *G. elata* on lists of endangered species [24]. Its cultivation and growth are difficult and often hindered by the presence of pathogenic fungi in the soil that can cause root rot, which also makes the use of pesticides necessary in some cases to avoid the loss of the plants or to allow the reuse of the same soil multiple times [25,26]. The alternative of using new soil for each cultivation cycle is not sustainable and increases costs; therefore, innovative techniques of removing pathogens that allow soil reuse are of great value. Electron beam treatment has been successfully used in the cultivation of *Panax ginseng*, with much higher root survival (81%) compared to that in the untreated reused soil (0%) and a similar rate to that in the virgin soil (78%) as well as comparable levels of ginsenosides in both cases [25].

For complex sample analysis, hyphenated high-performance thin-layer chromatography (HPTLC) is a versatile tool that can be used for simultaneous sample separation, allowing authenticity screening [27,28,29,30,31,32], non-target bioprofiling [33,34,35,36], as well as quantification of chemical marker compounds [37,38,39,40,41,42,43,44] from a wide variety of samples, including foods, plants, and other natural products. The combination of multiple analytical steps and identification of bioactivity by the same workflow makes hyphenated HPTLC a very sustainable technique and allows complex mixtures, such as plant extracts, to be studied in parallel in a much faster and straightforward way [45,46].

In this study, soil treatment with an electron beam, which removes the pathogenic fungi, but at the same time allows beneficial bacteria to thrive and promote plant growth [25], was applied to the cultivation of *G. elata.* The cultivation facility using the treated soil was adapted from that used for *P. ginseng,* and *G. elata* samples were harvested after two years in different time intervals. The chemical composition and bioactivity of five *G. elata* extracts obtained from dried and steam-treated tuber samples cultivated in a facility utilizing electron beam-treated soil were compared to two extracts from plants grown in the field. For their analysis, hyphenated HPTLC combined with multi-imaging and non-target effect-directed on-surface assays was used.

## 2. Results

### 2.1. Origin, Harvest, and Post-Harvest Processing of Samples

Seed propagation, cultivation, and trade of *Gastrodia elata* Blume (G) have been authorized by the Korean government under the “Law on Protection and Management of Wildlife” and comply with internationally endangered species resolution (Conf. 11.15 (Rev. CoP18). Samples were cultivated as described (Section 4.2) at Muju-Gun, Jeollabuk-Do, South Korea. Three harvest cycles of the rhizomes were performed over a period of twelve months, and seven different samples were obtained, starting by a facility harvest in April 2021, followed by simultaneous harvests from the facility and field in November 2021, and one last cycle also from both facility and field in April 2022. After each harvest, the samples were boiled in steam at 80 °C for 5 min, cut, and naturally dried in the sunshine. This post-harvest processing was relevant for sample storage. Samples were consecutively named G1 to G5 according to the harvest period. To account for intrasample variability, for the first harvest period, three samples G1.1, G1.2, and G1.3 were collected and stored individually. Each sample was cut into pieces and pulverized (visual characteristics in Table S1). For comparison, one *Panax ginseng* (Pg) sample was prepared accordingly. This medicinal plant well known from traditional medicine was selected since facility cultivation and soil treatment was adapted from the protocol used for Pg cultivation.

### 2.2. Selection of the Extractant

Traditionally, *G. elata* is consumed after boiling the roots in water. The water and the roots are consumed. Similar to this traditional use, two different types of extracts were prepared at 100 mg/mL using either 50% ethanol in bidistilled water (50% EtOH) or decoction in bidistilled water at 95 °C for 1 h, both followed by ultrasonication for 30 min. All extracts along with the Pg extract were applied on the HPTLC plate silica gel 60 and developed with a mixture of ethyl acetate–methanol–bidistilled water–formic acid, 7.3/1.25/1.0/0.45, *V*/*V*/*V*/*V*, adapted from [47]. Detection was performed at FLD 366 nm after derivatization with Natural Product reagent A, followed by PEG treatment for visualization of phenolic compounds (Figure S1a) and with 2-naphthol sulfuric acid reagent for visualization of saccharides (Figure S1b). Similar G profiles between the 50% EtOH extracts and the boiled water extracts were observed but with much higher amounts of extracted compounds (more intense bands) for the 50% EtOH extracts, which was selected for further analyses. As expected, the co-analysis and comparison with the Pg sample showed different chemical profiles between both plants and, as expected, the absence of gastrodin (*hR*_F_ 50) in Pg (Figure S1b). Gastrodin, vanillin and vanillyl alcohol reported as bioactive substances in *G. elata* tubers [11,48] were used as chemical marker compounds.

### 2.3. Comparative Chemical and Effect-Directed Profiling

**Figure 1:**
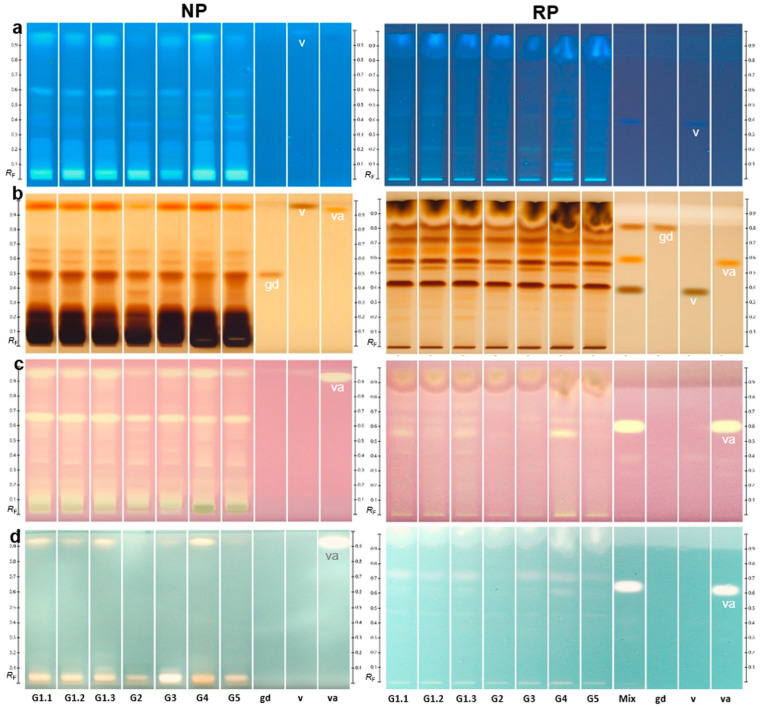
Chemical and effect-directed profiles of *Gastrodia elata* samples: G1–G5 (10 or 15 µg/band each) and chemical marker compounds gastrodin, vanillin and vanillyl alcohol (1 or 2 µg/band each) on HPTLC plates silica gel 60 developed with ethyl acetate–methanol–bidistilled water–formic acid (7.3/1.25/1.0/0.45, *V*/*V*/*V*/*V*) or HPTLC plates silica 60 RP-18 W developed with water–acetonitrile–methanol–formic acid (4.5/1/1/0.2, *V*/*V*/*V*/*V*), detected (**a**) at FLD 366 nm via the Fast Blue salt B reagent (not shown) followed by Natural Product reagent A and PEG on NP, or just at FLD 366 nm on RP, and at white light illumination after the (**b**) Fast Blue salt B reagent followed by 2-naphthol sulfuric acid reagent, (**c**) DPPH• assay, and (**d**) ABTS assay.

Four identical HPTLC silica gel 60 plates (NP) containing the seven G extracts (10 µg/band each) were prepared. For chemical derivatization, a reagent sequence was used with Fast Blue salt B (image under white light illumination not shown since not much was visible), followed by Natural Product reagent A and PEG for visualization of phenolics (Figure 1a) and the 2-naphthol sulfuric acid reagent mainly used for visualization of saccharides, among others (Figure 1b). For detection of antioxidants, the diphenyl-1-picrylhydrazyl (DPPH•) assay (Figure 1c) and the 2,2′-azino-di-(3-ethylbenzthiazoline sulfonic acid)(ABTS) assay (Figure 1d) were used. The autograms were recorded instantly. On the DPPH• autogram (Figure 1c), yellow bands on a purple background indicated radical scavenging activity. Apart from strong signals at the application zone, one prominent antioxidant band at *hR*F 67 was detected in all samples, and another band at *hR*F 95 was also detected, although weaker in the response for samples G2 and G5. In the ABTS autogram (Figure 1d), antioxidant components were observed as colorless (white) zones on a green background. Apart from strong signals at the application zone, the previous antioxidant band at *hR*F 95 was also detected by this assay, again weaker in the response for samples G2 and G5. The reference vanillyl alcohol showed a strong antioxidant response at a slightly lower *hR*F for both assays which needs further confirmation (shown subsequently). The acetylcholinesterase inhibition autogram did not show bands with strong activity at the same amounts applied (Figure S2). The very weak inhibition response at *hR*F 57 was slightly stronger for samples G2 and G5.

**Figure 2:**
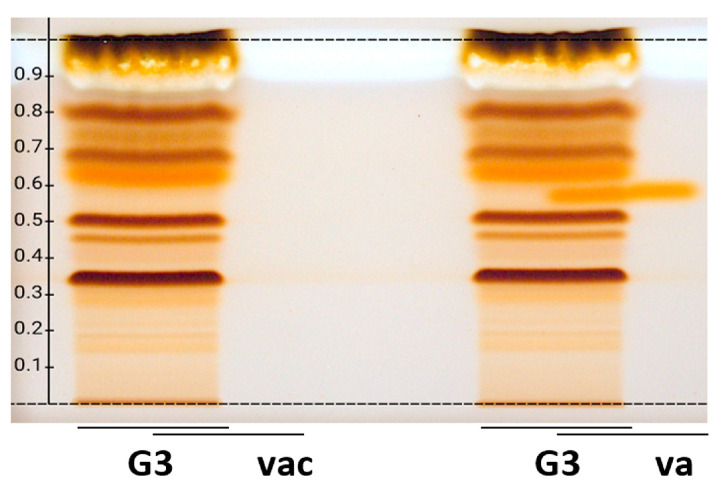
Overlapped application of the gastrodia sample extract (G3, 30 µL, 100 mg/mL in 50% ethanol) with the reference vanillic acid (vac, not detectable) and vanillyl alcohol (va; both 3 µL each, 1 mg/mL in methanol) applied as a 21 mm band each with a 7 mm overlapping part on HPTLC plates silica 60 RP-18 W developed with water–acetonitrile–methanol–formic acid (4.5/1/1/0.2, *V*/*V*/*V*/*V*), and detected at white light illumination after the 2-naphthol sulfuric acid reagent.

The results of the effect-directed profiling were confirmed on wettable reversed phase RP-18 W layers (Figure 1). An orthogonal mobile phase system had to be developed, and the extracts were successfully separated using water–acetonitrile–methanol–formic acid 4.5/1/1/0.2, *V*/*V*/*V*/*V*. The chemical marker compounds gastrodin, vanillin, and vanillyl alcohol were migrating in the reverse order, and the zone resolution between vanillin and vanillyl alcohol was substantially improved. Vanillin was not visible/detectable at the given amounts in the samples in the chromatograms/autograms. Vanillyl alcohol on the RP plate again showed an antioxidant response at a similar position as an antioxidant band in the samples for both assays. However, the *hR*F of vanillyl alcohol compared to the samples was not identical, which was proven by confirmative studies via overlapped application to study the retardation behavior of vanillyl alcohol partially migrating in the matrix (Figure 2). This showed that vanillyl alcohol did not match to the marker compound in the samples. The recording of high-resolution mass spectra [49] of this vanillyl alcohol-like compound zone showed the base peak at *m*/*z* 459.1149 [M-H]^–^ in the negative ionization mode, confirmed by the respective sodium adduct in the positive ionization mode, which was tentatively assigned to the molecular formula of C_19_H_24_O_13_. It was preliminarily assigned to Parishin E reported as a phenolic glycoside originally isolated from *G. elata* [50], however, needs further proof by co-chromatography. At least the chromatographic and spectral data as well as successful derivatization with the 2-naphthol sulfuric acid reagent underline this preliminary assignment.

**Figure 3:**
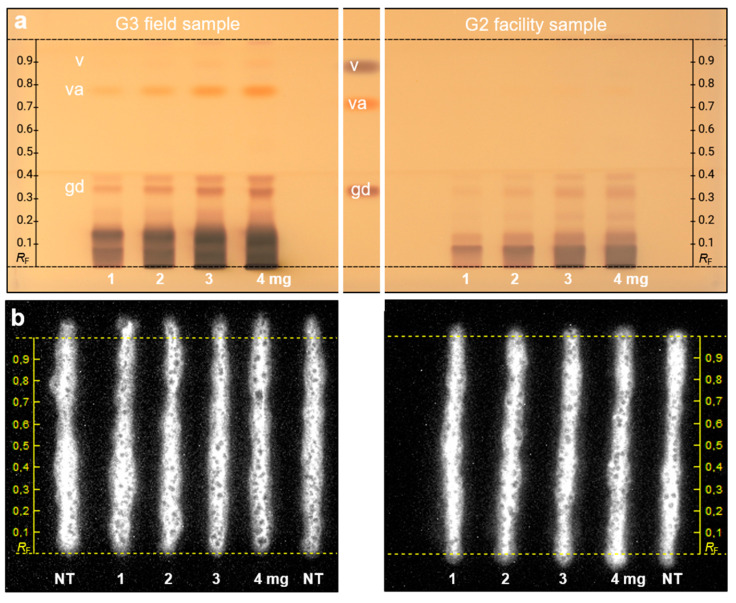
On-surface cytotoxicity bioassay via adherent HEK293T-CMV-ELuc cells: one facility (G2) and one field sample (G3) of *G. elata* (1–4 mg/band each, 10–40 µL/band) were applied on HPTLC plates silica 60 RP-18 W, two-step developed with (1) ethyl acetate–toluene (4/1, *V*/*V*) up to 6 cm, and after drying, with (2) ethyl acetate–methanol (4/1, *V*/*V*) up to 3 cm, and detected (**a**) at white light illumination after derivatization with the 2-naphthol sulfuric acid reagent and (**b**) cell bioluminescence after the cytotoxicity bioassay (depicted as a greyscale image; NT: non-treated cells applied on the plate background and used as negative control; plate with positive control in Figure S3c).

The latest cytotoxicity bioassay protocol for the adherent HEK 293T-CMV-ELuc cells on the HPTLC plate silica 60 RP-18 W [28] was used in the first experiment (Figure S3a without development). The *G. elata* G1.1 extract was manually applied along with a *Saussurea costus* extract (100 mg/mL methanolic solution [28]) used as plant-based positive cytotoxic control, both 500 µg/band, and curcumin (2 µg/band) as another example compound to be tested due to its strong light-absorbing pigments. Cytotoxicity of the samples was tested using the tetrazolium salt MTT to observe a reduction in purple-colored vivid cells and the luciferin solution to detect a reduction in cell bioluminescence. After a 24 h incubation, cytotoxicity was only observed for *S. costus*, but not for *G. elata.* Then, the samples were applied again and two-step separated, first with a comparatively more apolar solvent mixture (ethyl acetate–toluene 4/1, *V*/*V*) up to 6 cm, dried, and then developed with a more middle polar solvent mixture (ethyl acetate–methanol 4/1, *V*/*V*) up to 3 cm. The *G. elata* G1.1 and *S. costus* samples were compared, and again cytotoxicity was only observed for *S. costus* (Figure S3c). To confirm the absence of cytotoxicity for *G. elata*, another facility sample (G2) and one field sample (G3) from the same harvest were studied in a two-step development (Figure 3).

Each sample was applied twice at increasing amounts (1–4 mg/band). After the plate cut, one sample set was subjected to the derivatization and the other to the cytotoxicity bioassay. After derivatization with the 2-naphthol sulfuric acid reagent, the preliminary assigned Parishin E (*hR*F 78) was observed for G3 but not for G2, and the gastrodin at *hR*F 35 for G3 and less intense for G2 (Figure 3). HEK293T cells expressing luciferase were again used for the on-surface cytotoxicity assay, as previously described [28]. After the 24 h incubation, cytotoxicity was not observed for either G2 or G3, as evident from the intact bioluminescent HEK 293T-CMV-ELuc cell stripes on the increasing amounts of both samples tested. Even with the highest applied sample amount of 4 mg, which almost overloaded the adsorbent, no cytotoxic effect was observed.

### 2.4. Quantification of the Marker Compound Gastrodin

**Figure 4:**
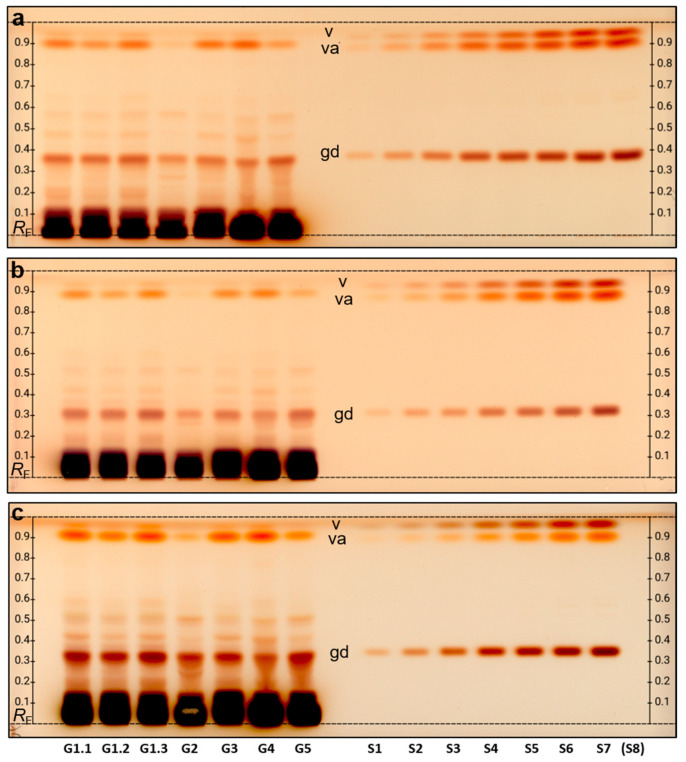
Quantification of chemical marker compounds: HPTLC-Vis profiles of seven *Gastrodia elata* samples applied at (**a**) 10 µL/band of 100 mg/mL, (**b**) 30 µL/band of 33 mg/mL, and (**c**) 60 µL/band of 33 mg/mL, along with different calibration levels S1–S8 of gastrodin (gd), vanillyl alcohol (va) and vanillin (v) (0.1, 0.2, 0.5, 1, 2, 3, 4 and 5 µg/band each) on HPTLC plates silica gel 60 developed with ethyl acetate–methanol–bidistilled water 7.3/1.25/1.0, *V*/*V*/*V*, and detected at white light illumination after derivatization with the 2-naphthol sulfuric acid reagent.

For quantification of the chemical marker compound gastrodin, the seven extracts of the G samples (10 µg each, 10 µL/band) were applied together with seven (or eight) different standard levels. The preliminary assigned Parishin E was equivalently calculated via the vanillyl alcohol signal response after derivatization, which was found to be comparable from the chromatographic and spectral properties. For separation of the preliminary assigned Parishin E from the front-eluting vanillin, the solvent strength of the mobile phase was reduced, i.e., the formic acid was removed but the proportions of the other solvents remained (ethyl acetate–methanol–bidistilled water (7.3/1.25/1.0, *V*/*V*/*V*). After derivatization with a 2-naphthol sulfuric acid reagent, the samples G4 and G5 showed band distortions. Such a matrix effect can be observed for samples with high contents of saccharides [37,43,51,52]. At *hR*F 30–40, it is noticeable that the band corresponding to gastrodin is distorted in samples G4 and G5 (Figure 4a), which normally leads to an increased quantification error. To circumvent this distortion, a known strategy in HPTLC is diluting the sample with the same solvent and applying a respective higher sample volume to obtain the same amount [53]. In this case, 30 µL of a 1:3 dilution of the 100 mg/mL extracts (Figure 4b) was applied.

Gastrodin quantification was successful under this condition; however, it was not yet possible to equivalently calculate the preliminary assigned Parishin E for samples G2 and G5, and thus double the amount of extract was applied (60 µL of the 1:3 dilution). Although this brought back the interference of the matrix for samples G4 and G5, the detection of the preliminary assigned Parishin E (*hR*F 90) was improved for samples G2 and G5 (Figure 4c). Hence, in a future plant extract screening, two different volumes of an unkown sample should be applied to allow for quantification. Due to the very low vanillin content in the sample and migration close to the solvent front (*hR*F > 95), the quantification of vanillin remained challenging, even for the higher amounts applied.

The quantification of gastrodin as well as the semi-quantification of the preliminary assigned Parishin E calculated equivalently to vanillyl alcohol were repeated on different days via absorbance measurement at 580 nm and the overall mean was calculated (Table 1). The precision values represent the method’s ruggedness and are therefore higher since different sample dilutions and sample application volumes were used as discussed. In the future, the sample concentration could be fixed to 0.3 mg/mL and a 15 µL sample volume could be applied on a slightly larger area (8 mm × 3.5 mm) to better spread the matrix at the application zone and thus improve its penetration by the mobile phase mixture during the development.

As observed for the HPTLC profiles and calculated mean content, sample G4 (facility sample from April 2022) had the lowest gastrodin content, followed by G2 (facility sample from November 2021). G2 was also the sample with the lowest preliminary assigned Parishin E content, but G4 was the one with the highest content. The two important field samples G3 and G5 were contrasting in the results; while G5 had a higher gastrodin content (by ¼ higher), it was half in the preliminary assigned Parishin E content compared to G3. In contrast, sample G1 showed a high content of both substances. Samples G1.1 and G1.3 had very similar results (both highest content of gastrodin and second highest content of the preliminary assigned Parishin E), whereas sample G1.2 showed a slightly lower content of gastrodin (by 15%) and even lower content for the preliminary assigned Parishin E (by ca. 40%). Hence, also within the same batch, there are differences to be expected between the individual tubers.

## 3. Discussion

Hyphenated HPTLC [45] and its miniaturization to an open-source 2LabsToGo system [54] is a straightforward, fast, and low-cost technique for the separation of complex mixtures. It contributes to more sustainable methods, analyzing all samples simultaneously with less consumption of chemical reagents. Up to twelve hyphenation dimensions including substance identification via high-resolution mass spectrometry have been reported for HPTLC [33,34]. Hence, to exploit the potential of hyphenated HPTLC, it was successfully applied for the first time to bioactivity screening of *Gastrodia elata* samples. The novel cultivation in the facility using electron-beam-treated soil and the inoculation of the symbiotic fungi allowed plant growth as for the cultivation in the field. As expected [26,55,56], differences between the seven studied facility and field samples and the different harvest times were observed. Variations in the number of radical scavenging bands among samples were observed, which can also have been influenced by the post-harvest processing of the different batches [57]. The prominent antioxidative zone at *hR*F 95 was assumed to be Parishin E, which still needs confirmation by co-chromatography. As the other prominent antioxidative zone at *hR*F 67 (Figure 1c, NP) did not match with the marker compound gastrodin, its identification through elution to high-resolution mass spectrometry might be interesting. In comparison to the antioxidative potential, the acetylcholinesterase inhibition response of the seven *G. elata* samples was very weak. Encouragingly for commercialization, none of the facility or field samples tested showed cytotoxic properties even at high amounts (4 mg) studied, which was close to overloading the adsorbent.

Exploiting microchemical derivatization for selective detection, the quantitative analysis of the chemical marker compounds gastrodin and further compounds was simply performed, contrary to complex instrumentation used otherwise [48,58]. Differences in the gastrodin content were evident. The preliminary assigned Parishin E was equivalently calculated to vanillyl alcohol based on the signal response obtained after derivatization, which was found to be comparable from the chromatographic and spectral behavior. The report of vanillyl alcohol as a chemical marker in the *G. elata* rhizome/tuber in the literature [11,48,59,60] is herewith questioned. Vanillin was not quantified since it was present at low amounts and too close to the solvent front. Thus, the present quantitative method still needs further improvement as mentioned.

The *G. elata* rhizome/tuber with attributed cytoprotective and other beneficial properties due to its chemical marker compounds [61,62,63,64,65,66,67] has a similar potential to that of *Panax ginseng,* which is an example of a traditionally used species that after many studies and confirmation of its beneficial properties has been spread around the world. After further optimization of the facility cultivation, *G. elata* is assumed to successfully provide high amounts of standardized extracts, rich in bioactive secondary plant metabolites [68,69,70].

## 4. Materials and Methods

### 4.1. Chemicals and Materials

HPTLC plates silica gel 60 (batch HX13161141) as well as HPTLC plates silica gel 60 RP-18 wettable (W) (batch HX28689296), all 20 cm × 10 cm, were obtained from Merck (Darmstadt, Germany). Before use, HPTLC plates silica gel 60 were pre-washed with methanol–water (4:1 *V*/*V*), dried in an oven (Memmert, Schwabach, Germany) for 20 min at 110 °C, and stored wrapped in aluminum foil. The RP-18 W layer binder was hardened by heating at 120 °C for 1 h (Plate heater, CAMAG). After cooling down, the plates were pre-washed first with methanol and then with ethyl acetate. All salts (per analysis quality) were water-free unless stated otherwise. All solvents were of high-performance liquid chromatography (HPLC) grade. Ethanol, methanol, ethyl acetate, Triton X-100, glycerol, vanillin, 2–aminoethyl diphenyl borate (natural product reagent A), sulfuric acid, and tris-(hydroxymethyl)-aminomethane (Tris) were obtained from Carl Roth (Karlsruhe, Germany). Acetic acid was purchased from VWR Chemicals (Radnor, PA, USA), and polyethylene glycol 6000 (PEG) was obtained from J.T. Baker-Avantor (Deventer, The Netherlands). Dulbecco’s Modified Eagle Medium (DMEM high glucose), DMEM/F12 medium without phenol red, fetal bovine serum, hygromycin B, and TrypLE Express solution were bought from Gibco (Carlsbad, CA, USA). Phosphate-buffered saline, ethylenediaminetetraacetic acid (EDTA), tricine, dithiothreitol (DTT), trans-1,2-cyclohexane diamine tetraacetic acid monohydrate (CDTA), citric acid, all salts for buffer preparations, thiazolyl blue tetrazolium bromide (MTT), Fast Blue salt B, 2-naphthol, 2,2′-azino-di-(3-ethylbenzthiazoline sulfonic acid) (ABTS), potassium persulfate and penicillin/streptomycin solution for cell culture were obtained from Sigma-Aldrich (Steinheim, Germany). Magnesium carbonate hydroxide pentahydrate and diphenyl-1-picrylhydrazyl (DPPH•, 95%) were bought from Alfa Aesar (Karlsruhe, Germany). Double-concentrated phosphate-buffered saline was obtained from Biochrom (Berlin, Germany). D-Luciferin sodium salt and adenosine triphosphate were purchased from Cayman Chemical Company (Ann Arbor, MI, USA). Bidistilled water was prepared using a Heraeus Destamat Bi-18E (Thermo Fisher Scientific, Schwerte, Germany). Gastrodin was obtained from Phytolab (Vestenbergsgreuth, Germany). Vanillyl alcohol was obtained from Acros Organics (ThermoFisher Scientific, Geel, Belgium). The origin of HEK293T cells constitutively expressing enhanced beetle luciferase (ELuc) was previously described [28].

### 4.2. Cultivation, Harvest, and Post-Harvest Processing

*Gastrodia elata* Blume samples were cultivated at 150-91 Domaro Mupung-Myeon, Muju-Gun, Jeollabuk-Do, Republic of Korea. The mature rhizomes, disease free, and with an evident flowering shoot at the end were planted in sand soil. The pots were maintained in the dark at 24 °C. After six weeks, manual pollination was conducted as the flowers opened. Each tuber produced very tiny seeds (ca. 14 µg per 4 million seeds, 1.0 mm in length, and 0.5 mm in width). Capsules with mature seeds were harvested about three weeks after manual pollination before its dehiscence and stored at 4 °C. The two symbiotic fungi *Mycena osmundicola* and *Armillaria mella* were obtained from the Muju Rural Technology Center for seed germination. The mycelia culture medium was produced by mixing rice bran and distilled water. Homogenized inoculum of symbiotic fungi was applied to mycelia culture and mixed with fallen leaves and branches of *Quercus* species for 4 weeks. The seeds of *G. elata* were germinated by the orchid mycorrhizal fungus nutrition of *Mycena osmundicola*. During 8 weeks of symbiotic culture, the initiation of the protocorm mainly elongated up to about a 6 mm development was induced. Subsequent infection with *Armillaria mella* allowed the protocorm to further develop into juvenile tubers grown to approximately 50 mm at 24 °C in the dark for 16 weeks. The immature tubers were directly transplanted for production in the field or facility, embedded with fallen leaves and branches of *Quercus* species inoculated with *Armillaria mella*. One hundred tubers were planted per square meter, cultivated, and grown to a size bigger than 100 mm for 1 year. In the Korean facility, irrigation was performed with sprinklers and fog water systems. The field soil was irrigated by rainfall and artificial sprinklers. The facility is an interlocking panel-type house with smart multi-stage cultivation and natural lighting. The harvest dates, after two years of cultivation, were 1 April 2021, 1 November 2021, and 1 April 2022. The post-harvest process included boiling in a steam of 80° C for 5 min and natural drying in sunshine.

### 4.3. Extraction and Standard Solutions

Each sample was milled at 25,000 rpm for two rounds for 1 min using a small laboratory grinder (Tube-Mill control, IKA, Staufen, Germany). Two types of extracts were prepared for each sample, using either 50% ethanol in bidistilled water or only bidistilled water digerated at 95 °C for 1 h. Each ground sample (300 mg) was placed inside a centrifuge tube and vortexed with a 3 mL extractant for 30 s, followed by ultrasonication for 30 min (Sonorex Digiplus, Bandelin, Berlin, Germany). After centrifugation at 3000× *g* for 10 min (Labofuge 400, Heraeus, Hanau, Germany), the supernatants were transferred to sampler vials (100 mg/mL). Gastrodin, vanillin, and vanillyl alcohol standard solutions were prepared as 1 mg/mL solutions in methanol and transferred to a sampler vial.

### 4.4. HPTLC–UV/Vis/FLD Method

Extracts (0.1 µL/band) were applied as 7 mm bands on a pre-washed plate (Automatic TLC Sampler 4, CAMAG, Muttenz, Switzerland). For higher application volumes (10–60 µL/band), the samples were applied as an area (7 mm × 3.5 mm). Calibration standards (gastrodin, vanillin, and vanillyl alcohol, 0.2–5 µL/band each) were applied for quantification. Plates were developed with ethyl acetate–methanol–bidistilled water–formic acid (7.3/1.25/1.0/0.45 *V*/*V*/*V*/*V*) for the bioactivity profiling, or with ethyl acetate–methanol–bidistilled water (7.3/1.25/1.0, *V*/*V*/*V*) for quantification, both up to a migration distance of 7 cm in a twin trough chamber (20 cm × 10 cm, CAMAG). After plate drying for 4 min with a stream of cold air (hair dryer), the plates were documented at Vis and FLD 366 nm (TLC Visualizer 2, CAMAG). For derivatization, the following reagent sequence was applied through automatic piezoelectric spraying (Derivatizer, CAMAG), i.e., first, Fast Blue salt B solution (100 mg Fast Blue salt B in 100 mL 70% ethanol, freshly prepared) followed by intermediate drying was applied, then either the Natural Product A reagent (1 g 2–aminoethyl diphenyl borate in 100 mL ethanol) followed by a PEG solution (6% polyethylene glycol 6000 in ethanol) and plate drying followed by documentation, or the 2-naphthol sulfuric acid reagent (5 g 2-naphthol in 33 mL of ethanol followed by 20 mL of sulfuric acid added dropwise, and finally 127 mL of ethanol and 13 mL of water) heated at 130 °C for 5 min followed by documentation was applied. For quantification, absorbance measurement was performed at 580 nm (TLC Scanner 4, CAMAG). Mainly polynomial regressions were used for building the calibration curves (Figures S4 and S5). The software visionCATS (version 3.2.22308.1, CAMAG) operated and controlled the instruments. The recording of high-resolution mass spectra was performed as reported [49].

### 4.5. Bioactivity Profiling

The antioxidant DPPH• assay [71] was performed by spraying 4 mL of 0.04% methanolic DPPH• solution (green nozzle, level 4). The antioxidant ABTS·assay [72] was performed by immersing the plate in 50 mL ABTS·solution (1:1 mixed freshly from 7 mmol/L of the diammonium salt solution and 2.45 mmol/L K_2_S_2_O_8_ solution) for 2 s (TLC Immersion Device, CAMAG), followed by drying in the ambient air for 30 s.

For the acetylcholinesterase inhibition assay [46], the plates were pre-wetted with a 0.5 mL TRIS-HCl buffer (7.55 mg/mL TRIS, pH 7.8 adjusted with HCl, green nozzle, level 6). Then, a 1.5 mL acetylcholinesterase solution (6.66 U/mL plus 1 mg/mL BSA in TRIS-HCl buffer) was applied (green nozzle, level 6). The plate was incubated at 37 °C for 30 min. For detection, a 0.5 mL substrate–chromogenic reagent solution (1 mg/mL indoxyl acetate and 2 mg/mL Fast Blue salt B in ethanol) was sprayed (2 mL, green nozzle, level 6) to obtain colorless (white) inhibition zones on a purple background. The positive control was rivastigmine (0.1 mg/mL in methanol, 2, 4, and 8 μL/band).

For the cytotoxicity bioassay [28], HEK 293T-CMV-ELuc cells were cultivated, harvested from the culture flasks, and resuspended in assay medium DMEM/F12 without phenol red supplemented with a 5% fetal bovine serum solution and penicillin/streptomycin. Before application of the cells, RP-18 W plates were neutralized by immersion in a citrate buffer solution of pH 12 (6 g/L citric acid monohydrate and 10 g/L of disodium hydrogen phosphate anhydrate), followed by plate drying, immersion in double concentrated phosphate-buffered saline (9.55 g in 500 mL bidistilled water), and removal of the liquid excess. Cell application was performed as a stripe along each track. Plate incubation for 24 h followed. The bioassay [28,49] was slightly modified, using the application of 400 µL cell suspension (containing 5000 cells/µL) and an adhesive tape for sealing of the incubation chamber. For detection of the cell bioluminescence, the plate was completely dried under cold air (hair dryer) and immersed (immersion speed 3 cm/s, immersion time 5 s) twice into the luciferin solution (40 mM tricine, 2.14 mM magnesium carbonate hydroxide pentahydrate, 5.34 mM magnesium sulfate heptahydrate, 0.2 mM EDTA, 3 mM DTT, 1.1 mM D-luciferin and 20 mM adenosine triphosphate and mixed with lysis buffer containing 25 mM Tris pH 7.8, 2 mM DTT, 2 mM CDTA, 1% Triton X-100 and 10% glycerol, and citrate buffer pH 12). The bioluminescence was recorded using exposure times of 1 and then 10 min (Bioluminizer, CAMAG). In addition, the tetrazolium salt MTT was used (Figure S3a) to detect cytotoxicity as described elsewhere [28].

## Figures and Tables

**Table 1:** Method ruggedness: Quantification of the mean gastrodin contents (± standard deviation, sd) in the *G. elata* samples via calibrations performed on 3 to 5 days and plates with different sample dilutions/volumes applied; semi-quantification of the preliminary assigned Parishin E equivalently calculated to vanillyl alcohol.

Method Ruggedness: Mean Contents (*n* = 3–5 days/plates; ±Standard Deviation)		
*G. elata* Samples	Gastrodin (µg/100 mg ± sd)	Preliminary Assigned Parishin E (µg/100 mg ± sd) Equivalently Calculated to Vanillyl Alcohol
G1.1	209 ± 22	203 ± 19
G1.2	182 ± 16	129 ± 19
G1.3	218 ± 28	218 ± 14
G2	102 ± 27	24 ± 3
G3	146 ± 20	186 ± 10
G4	93 ± 11	235 ± 16
G5	198 ± 22	92 ± 6

## Data Availability

All data are available upon request.

## Supplementary Materials

The supporting information can be downloaded at: https://www.mdpi.com/article/10.3390/ijms24129936/s1.
