# Supplementary material for: Bioactivity Profiling and Quantification of Gastrodin in Gastrodia elata Cultivated in the Field versus Facility via Hyphenated High-Performance Thin-Layer Chromatography
Source: Int J Mol Sci. 2023 Jun 9;24(12):9936. doi: 10.3390/ijms24129936 (PMC10298505; doi:10.3390/ijms24129936)
Supplement: Supplementary file 1 [file ijms-24-09936-s001.zip › ijms-2360622-supplementary.pdf]

Supplementary materials

Bioactivity profiling and quantification of gastrodin in *Gastrodia elata* cultivated in the field *versus* facility via hyphenated high-performance thin-layer chromatography

Fernanda L.B. Mügge[1, #], Cheul Muu Sim[2], Bernd Honermeier[3] and Gertrud E. Morlock[1,*, #]

[1] Department of Food Science, Justus Liebig University Giessen, Giessen, Germany

[2] Neutron Science Center, Korea Atomic Energy Research Institute, Daejeon, Republic of Korea

[3] Department of Agronomy and Crop Physiology, Justus Liebig University Giessen, Giessen, Germany

[#]Both authors contributed equally.

***Correspondence: gertrud.morlock@uni-giessen.de**

Table S1 *Gastrodia elata* samples investigated

**April 2021**	
**Visual characteristics**	Pulverized sample
**G1.1 Facility sample**	
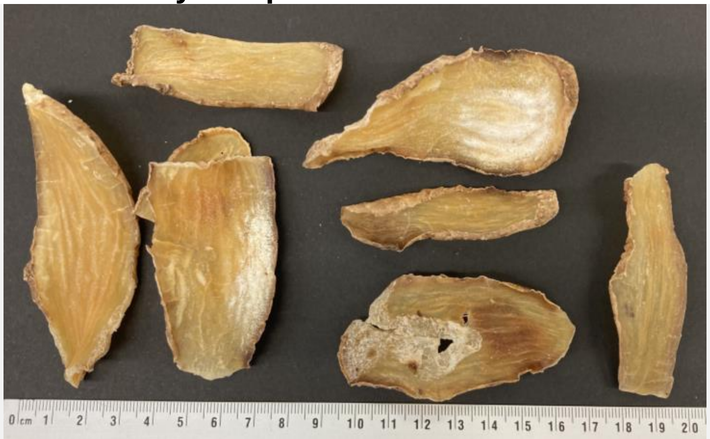	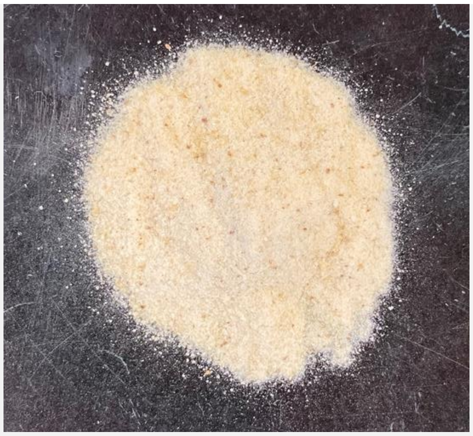
**G1.2 Facility sample**	
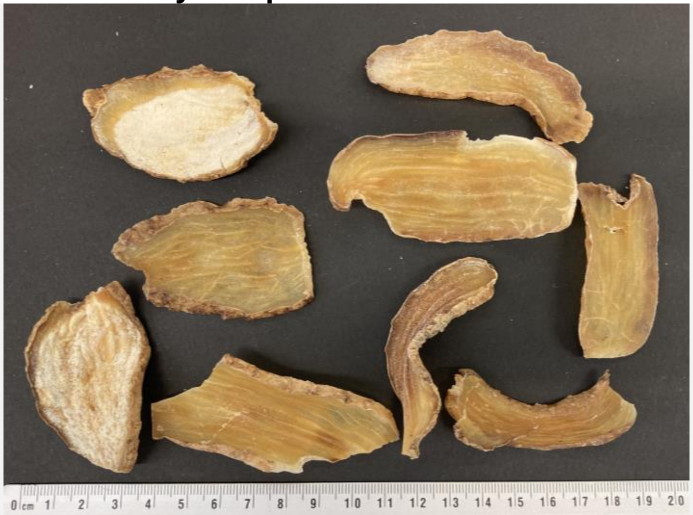	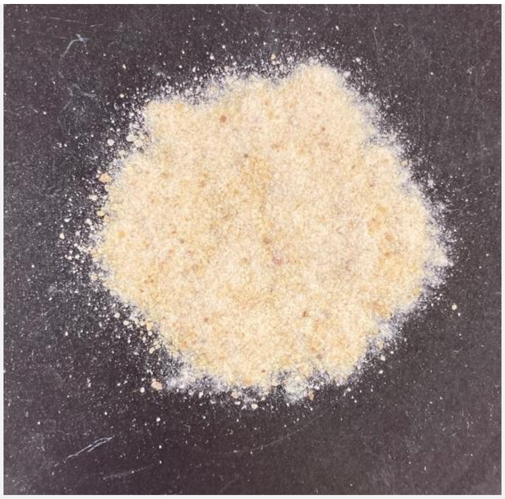
**G1.3 Facility sample**	
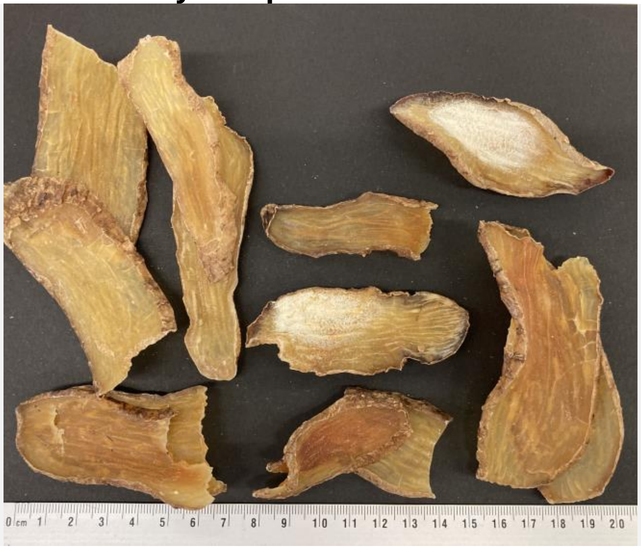	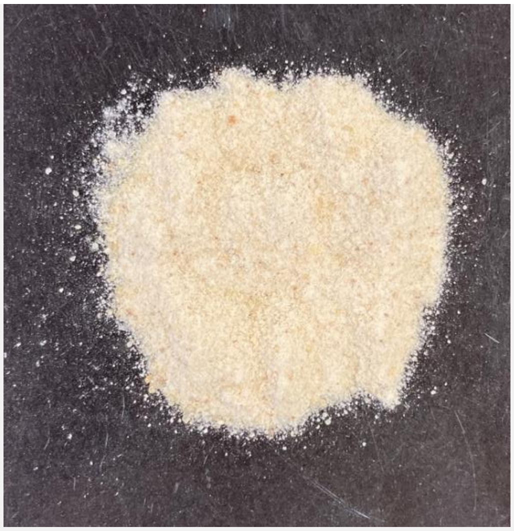

Table S1 continued

November 2021	
**Visual characteristics**	Pulverized sample
**G2 Facility sample**	
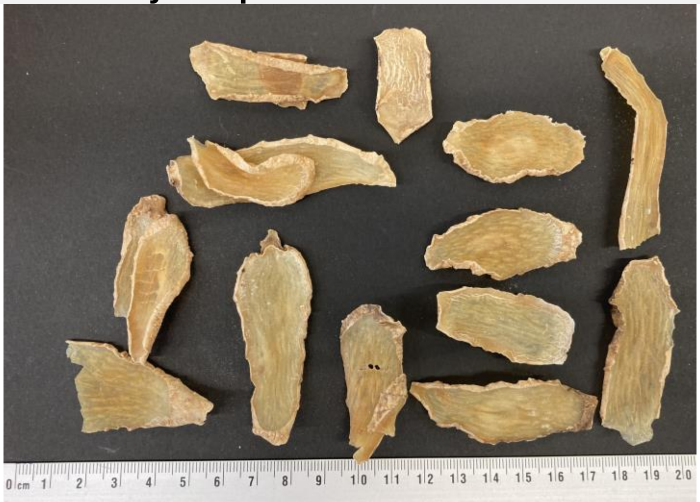	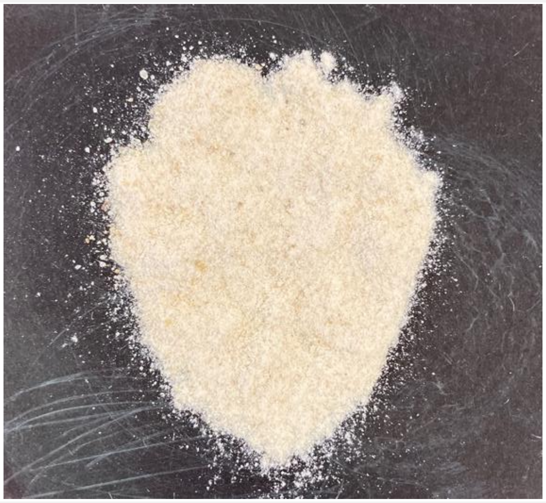
**G3 Field sample**	
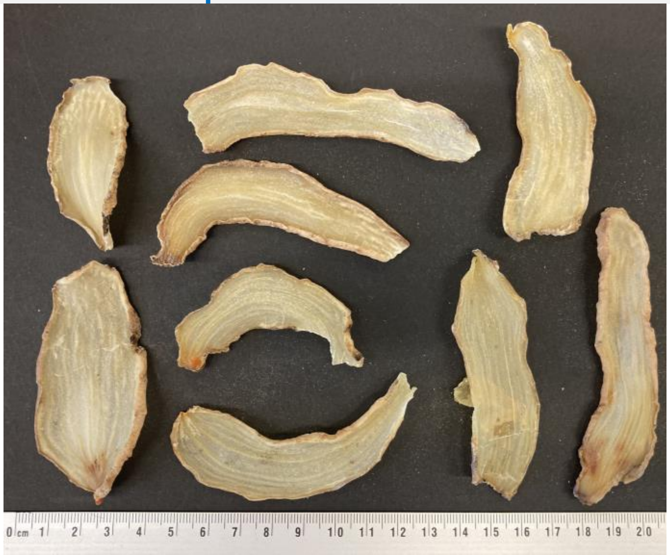	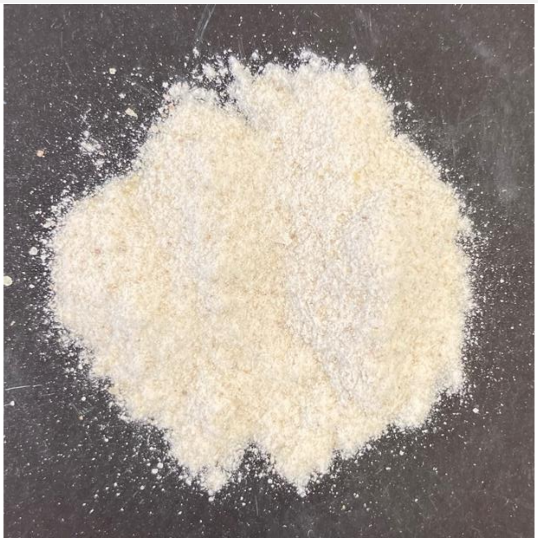

Table S1 continued

**April 2022**	
**Visual characteristics**	Pulverized sample
**G4 Facility sample**	
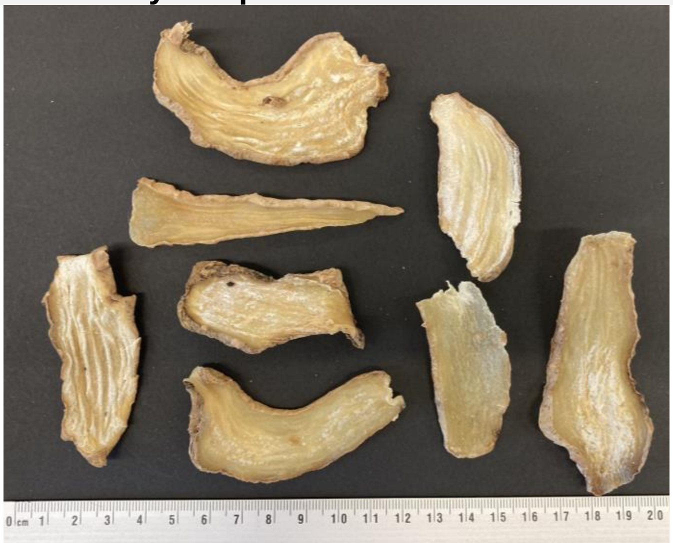	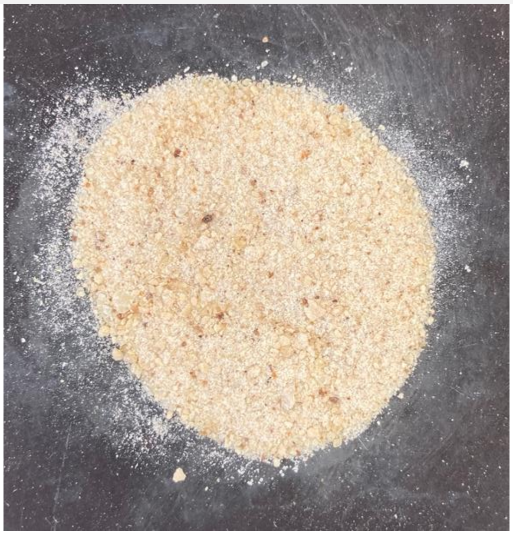
**G5 Field sample**	
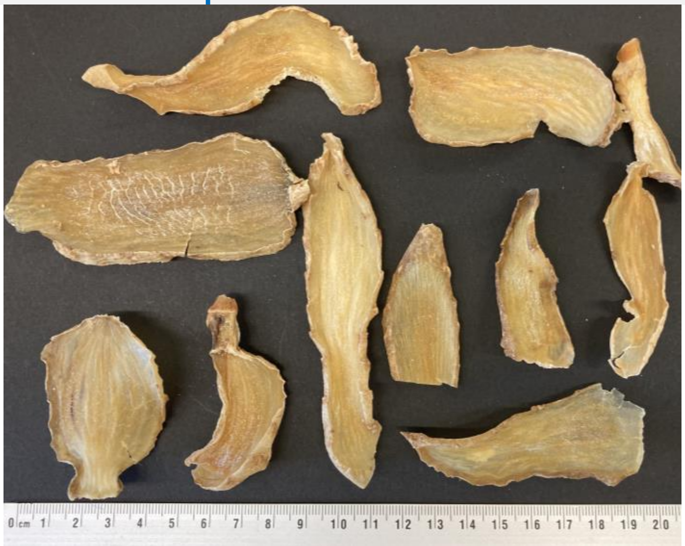	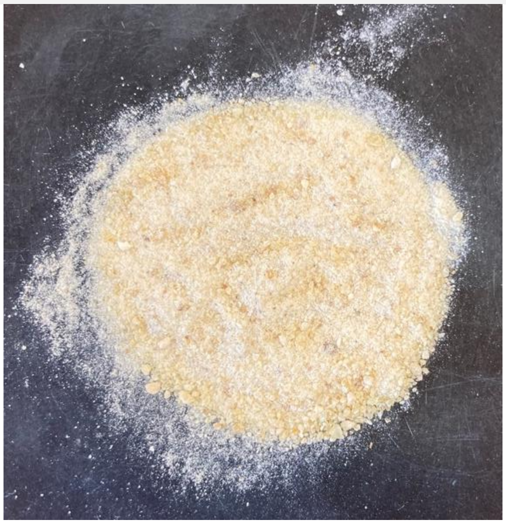

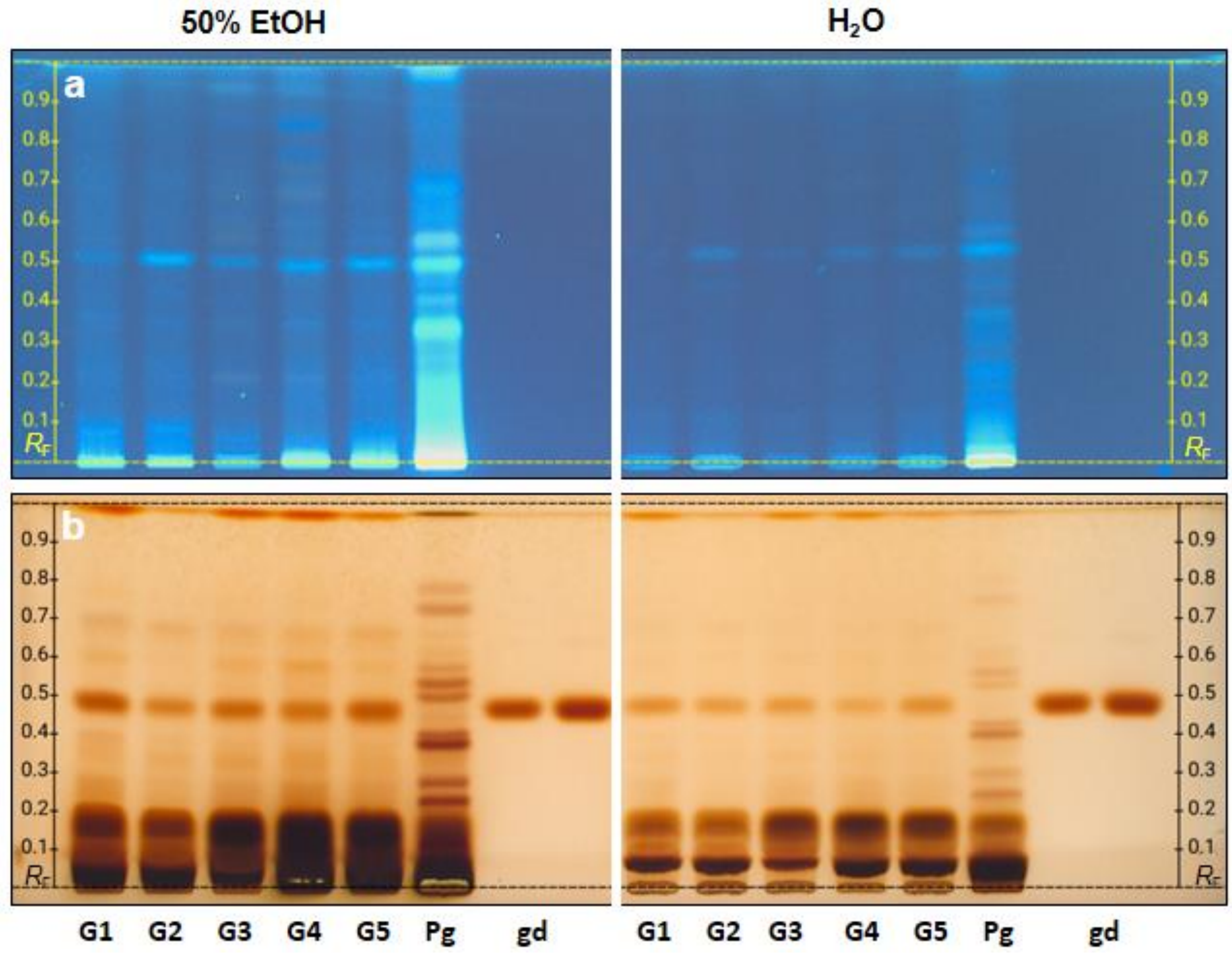

Figure S1 Comparative chemical HPTLC profiles of *Gastrodia elata* (G1–G5) and *Panax ginseng* (Pg) samples cultivated in the facility (G1, G2 and G4) or field (G3 and G5) were extracted with different extractants (100 mg/mL) either with 50% ethanol (EtOH, 12.5 µL/band) in bidistilled water or in bidistilled water at 95 °C (H_2O, 25 µL/ band) and applied along with gastrodin standard (gd, 2 and 4 µg/band, 2 and 4 µL of 1 mg/mL in methanol) on HPTLC silica gel 60 plates developed with a mixture of ethyl acetate – methanol – bidistilled water – formic acid (5.8/1.0/0.8/0.3) and detected at (**a**) FLD 366 nm after derivatization with the Natural Product A reagent followed by PEG and (**b**) white light illumination after derivatization with the 2-naphtol sulfuric acid reagent.

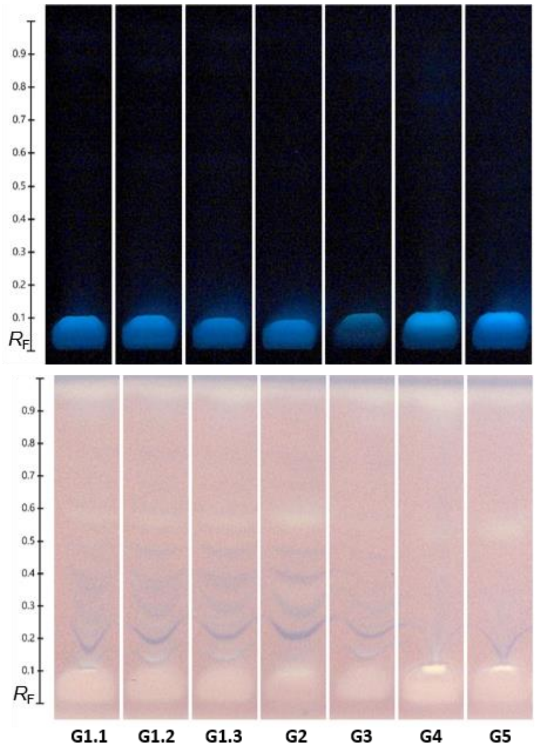

Figure S2. HPTLC chromatogram at FLD 366 nm and acetylcholinesterase inhibition autogram of *Gastrodia elata* samples G1–G5 (10 µg/band each, 10 µL) on HPTLC plates silica gel 60 developed with ethyl acetate – methanol – bidistilled water – formic acid (7.3/1.25/1.0/0.45, *V*/*V*/*V*/*V*), detected at white light illumination after the acetylcholinesterase inhibition assay.

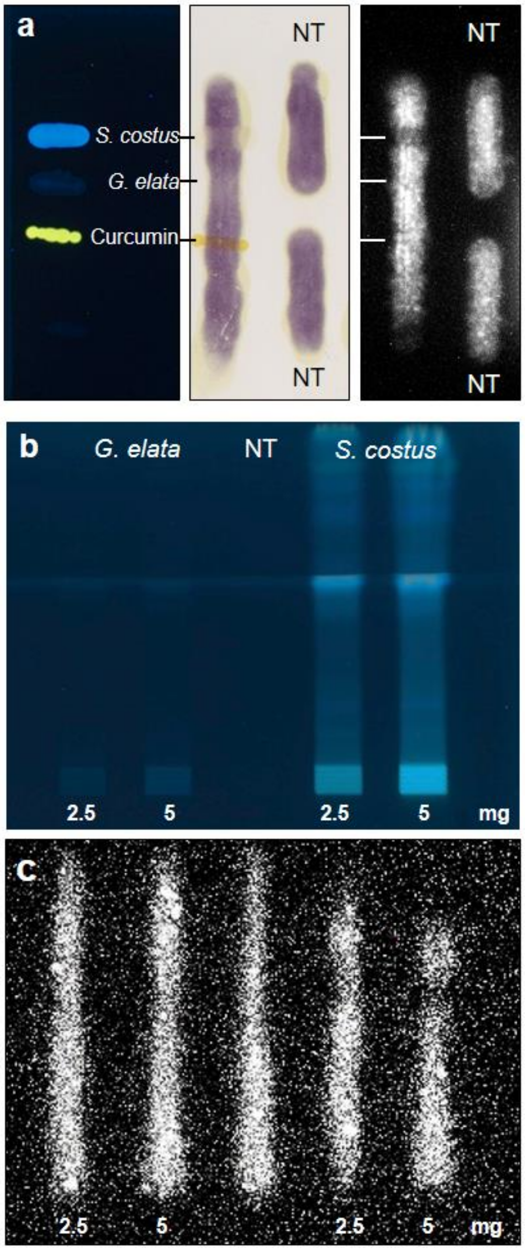

Figure S3 Initial cytotoxicity screening using adherent HEK293T-CMV-ELuc cells: *Gastrodia elata* (G1.1) and *Saussurea costus* extract samples [28] applied on HPTLC plates silica 60 RP-18 W (**a**) manually as band (no separation; both 500 µg/band, 5 µL of 100 mg/mL solution) along with curcumin (2 µg/band, 2 µL of 1 µg/mL solution), dried, cells applied on top (NT: two stipes of not treated cells applied on plate background used as negative control) and cytotoxicity detection either after MTT application by signal reduction of the purple MTT color or after luciferin application solution by reduction of the bioluminescence (as evident for *S. costus*); (**b**) as band (2.5 and 5 mg of *G. elata* or *S. costus*), followed by two-step development first with ethyl acetate – toluene (4/1, *V*/*V*) up to 6 cm, then after drying, with ethyl acetate – methanol (4/1, *V*/*V*) up to 3 cm, detected at FLD 366 nm and (**c**) after bioluminescence bioassay (depicted as a greyscale image), showing the cytotoxicity of *S. costus* but not of *G. elata*.

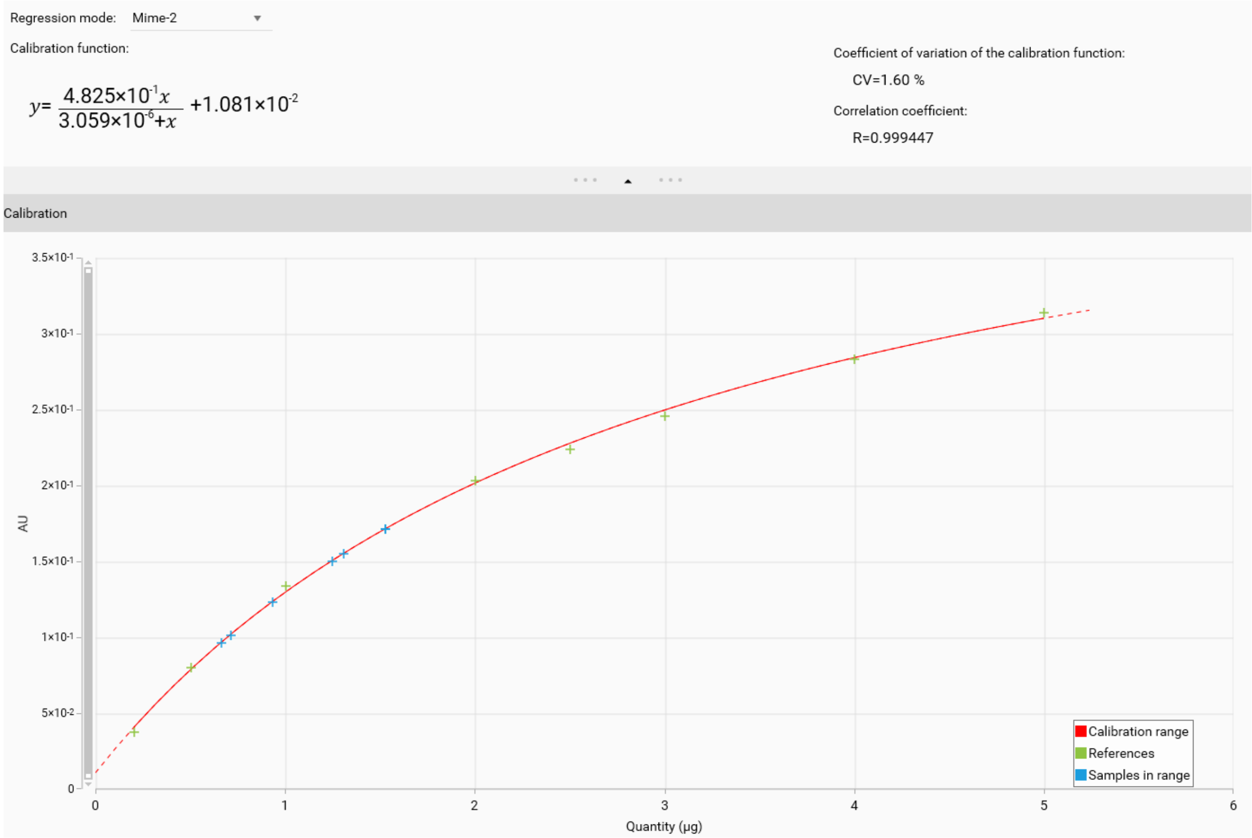

Figure S4 Example calibration curve of gastrodin at 580 nm (absorbance measurement)

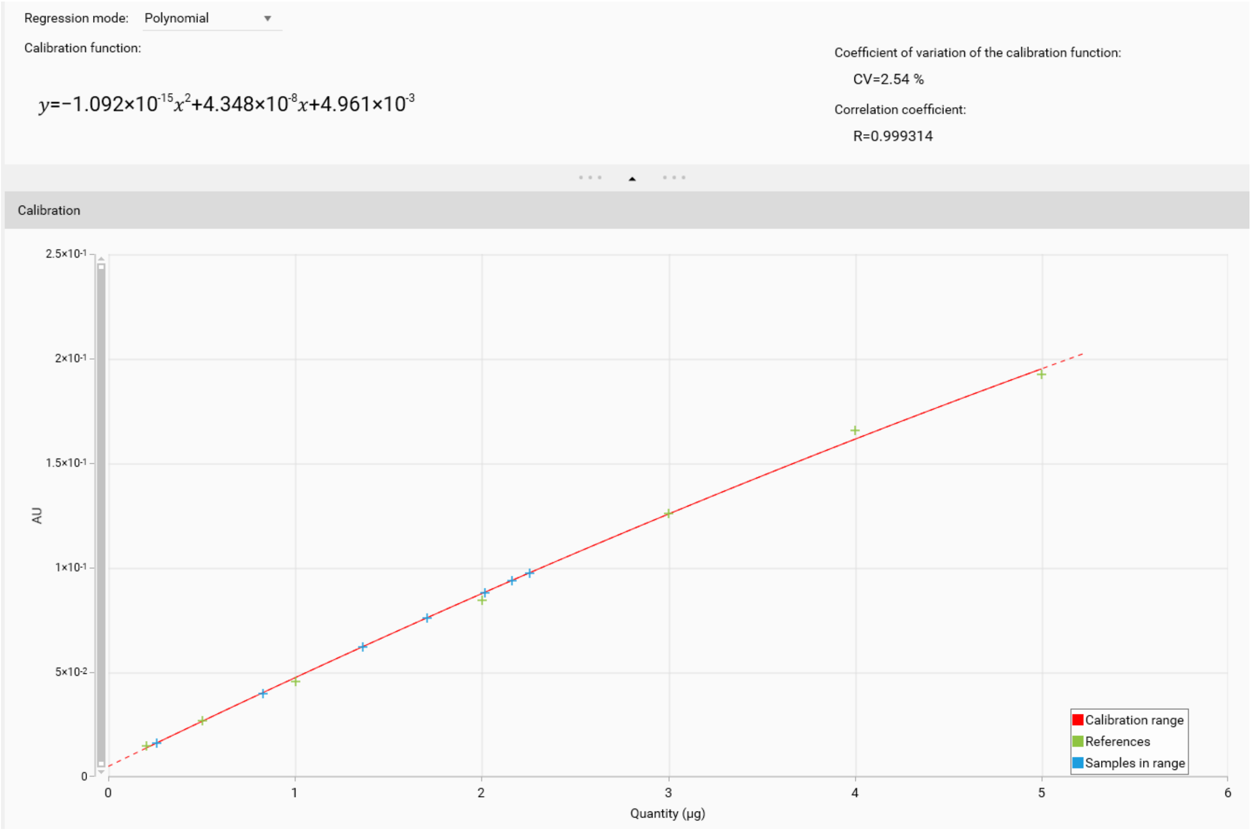

Figure S5 Example calibration curve of vanillyl alcohol at 580 nm (absorbance measurement)
